# AKR1B10 as a Potential Novel Serum Biomarker for Breast Cancer: A Pilot Study

**DOI:** 10.3389/fonc.2022.727505

**Published:** 2022-02-24

**Authors:** Zhe Cao, Kristin Delfino, Vivek Tiwari, Xin Wang, Abdul Hannan, Fawwad Zaidi, Andrew McClintock, Kathy Robinson, Yun Zhu, John Gao, Deliang Cao, Krishna Rao

**Affiliations:** ^1^ Hunan Cancer Hospital and The Affiliated Cancer Hospital of Xiangya School of Medicine, Central South University, Changsha, China; ^2^ Department of Medical Microbiology, Immunology and Cell Biology, Simmons Cancer Institute, Southern Illinois University School of Medicine, Springfield, IL, United States; ^3^ Center for Clinical Research, Southern Illinois University School of Medicine, Springfield, IL, United States; ^4^ Dartmouth Hitchcock Medical Center, Lebanon, NH, United States; ^5^ Division of Hematology/Medical Oncology, Department of Internal Medicine and Simmons Cancer Institute, Southern Illinois University School of Medicine, Springfield, IL, United States; ^6^ Southern Illinois University School of Medicine, Springfield, IL, United States; ^7^ Department of Pathology, Memorial Medical Center, Springfield, IL, United States

**Keywords:** AKR1B10, biomarker, breast cancer, serum, clinical trial

## Abstract

**Background:**

Aldo-keto reductase 1B10 (AKR1B10) is a secretory protein that is upregulated in breast cancer.

**Objective:**

This case-controlled pilot study evaluated the serum level of AKR1B10 in healthy women and patients with a localized or metastatic breast cancer.

**Methods:**

AKR1B10 levels were measured by ELISA and IHC in several patient cohorts.

**Results:**

Our data showed that serum AKR1B10 was significantly elevated in patients with localized (6.72 ± 0.92 ng/ml) or metastatic (7.79 ± 1.13 ng/ml) disease compared to cancer-free healthy women (1.69 ± 0.17 ng/ml) (p<0.001); the serum AKR1B10 was correlated with its expression in tumor tissues, but not with the tumor burden, molecular subtypes or histological stages. After surgical removal of primary tumors, the serum AKR1B10 was rapidly decreased within 3 days and plateaued at a level similar to that of healthy controls in most patients. ROC curve analysis suggested the optimal diagnostic cut-off value of serum AKR1B10 at 3.456 ng/ml with AUC 0.9045 ± 0.0337 (95% CI 0.8384 – 0.9706), sensitivity 84.75% (95% CI 73.01% to 92.78%), and specificity 93.88% (95% CI 83.13% to 98.72%).

**Conclusions:**

These data indicate the potential value of serum AKR1B10 as a biomarker of breast cancer.

## Introduction

Over 230,000 new invasive breast cancer cases are diagnosed in the United States annually, of which approximately 67% are in need of adjuvant chemotherapy treatment (1). Through decades’ efforts, therapeutic targets of breast cancer, such as estrogen receptor (ER) for tamoxifen treatment, have been identified and well characterized (2), but prevalent serum markers are still lacking thus far (3, 4). The currently used serum markers in breast cancer include Cancer Antigen 27.29 (CA 27.29) and CA 15.3; Rarely CEA has been used in the absence of elevated CA 27.29 and CA 15.3 (5). However, the sensitivity and specificity of these markers are considered low (6, 7). For instance, serum CA 27.29 is also increased in pregnancy, lactation and inflammatory diseases, as well as in ovarian, lung and prostate cancers (6, 8). Likewise, serum CA 15.3 levels are elevated in megaloblastic anemia, vitamin B12 deficiency, sickle cell disease and thalassemias. The American Society of Clinical Oncology (ASCO) does not recommend using CA 27.29, CA 15-3 or CEA for screening, diagnosis, and staging of breast cancer, or for monitoring response to treatment or recurrence after primary breast cancer therapy (8). CYFRA 21-1, a serum marker of lung cancer (9), is found to be elevated in metastatic breast cancer patients (10), but is not yet recommended by ASCO as a serum marker of breast cancer (8). Thus an urgent need exists for identification and validation of novel serum markers for breast cancer across the spectrum of disease, from screening and early detection to response monitoring during treatment.

Aldo-keto reductase 1B10 (AKR1B10) is a secretory protein that is primarily expressed in human colon and small intestine, but induced in hepatocellular carcinoma (HCC) (11), pancreatic cancer (12), and non-small cell lung cancer (13). AKR1B10 protein functions as an oncogenic protein that promotes tumor development and progression through elimination of cytotoxic carbonyl compounds (14–16) and elevation of lipogenesis (17, 18). AKR1B10 may also induce cell resistance to anthracyclines (19). In breast cancer, AKR1B10 protein is overexpressed in primary, metastatic, and recurrent diseases; AKR1B10 was also found to be upregulated in ductal carcinoma *in situ* (DCIS) (71.4%, n=28) (20). More interestingly, AKR1B10 is found to promote tumor growth in the metastatic sites and distant relapse of breast cancer through inhibition of the oxidative lesions derived from fatty acid oxidation (21). In fact, AKR1B10 activates diacylglycerol (DAG)-mediated PKC/ERK signaling in breast cancer cells (22) and promotes breast cancer metastasis *via* an integrin α5 and δ-catenin mediated mechanism (23). Similarly, AKR1B10 promotes brain metastasis of lung cancer (24). In prostate cancer, AKR1B10 may serve as an indicator of early relapse and radioresistance (25). In addition, AKR1B10 may provoke pancreatic cancer through mediation of K-Ras protein prenylation and cell apoptosis (12, 26), but in gastric cancer, AKR1B10 was reported as a favorable prognostic marker (27). The later was challenges by a recent report in which AKR1B10 was expressed in 58.5% gastric cancer and associated with a poor 5-years overall survival (28). Recently, it has been found that AKR1B10 is a secretory protein and is secreted through a secretory lysosome-mediated non-classical pathway, thus being a potential serum biomarker of breast cancer (20, 29). Heat shock protein 90α (HSP90α) mediates the cellular transportation and secretion of AKR1B10 protein (30). AKR1B10 is also upregulated in hepatocellular carcinoma and serves as a diagnostic marker (31). In mice, targeted disruption of AKR1B8, an orthologue of human AKR1B10, leads to abnormalities of intestinal epithelial self-renewal and high susceptibility to dextran sulfate sodium (DSS)-induced colitis and associated tumorigenesis (32).

In this case-controlled pilot study, we evaluated the serum levels of AKR1B10 in healthy women and patients with a localized and metastatic breast cancer. We estimated the dynamics of serum AKR1B10 before and after surgical removal of primary tumors and the serum fluctuation in response of metastatic treatment. We also evaluated the diagnostic values of the serum AKR1B10, and our data suggested the potential of serum AKR1B10 as a novel biomarker of breast cancer.

## Patients and Methods

### Ethics Statement

IRB approval of the protocols was obtained from Springfield Committee for Research Involving Human subjects (SCRIHS) and informed consent was obtained from all subjects prior to any study related activities. Procurement of serum and tissues occurred through two protocols: The Tumor Bank of Simmons Cancer Institute at SIU School of Medicine and SCRIHS protocol 11-118, specifically created for collection of prospectively collected serum and tissue specimens for this AKR1B10 research.

### Human Subject Enrollment

This study enrolled three cohorts of subjects from November of 2015 through July 2017. Cohort A consisted of women presenting to St. John’s Hospital for a screening mammogram that was subsequently confirmed to be normal and have no prior history of breast malignancy. Serum samples were collected from these women on the day of their mammogram. Cohort B consisted of patients presenting with a new breast cancer that had not metastasized distantly with the primary treatment modality being surgery. Specimens procured from Cohort B included serum, breast cancer tissues, and matched normal adjacent tissues to evaluate AKR1B10 expression in serum and primary tumors. Paraffin-embedded sections were also collected for immunohistochemistry if the availability of frozen tissues was limited. The matched serum samples were collected before/at the time of surgery and at various time points after the surgery (i.e., 3, 7 and 30 day’s post-surgery). Cohort C encompassed patients with metastatic or recurrent/relapsed breast cancer. All patients had their serum collected at the time of study entry and then serially monitored every three months for a period of two years. Tumor imaging data for overall tumor volumes (as measured by RECIST criteria) were collected on patients according to their normal routine care schedule, from which tumor burden was determined.

All subjects enrolled in Cohorts A-C were ≥ 18 years of age and had exclusions of active liver disease and chronic renal disease. Cohorts B and C had an exclusion of concurrent second-non-breast malignancy. Specific cohort eligibility was as follows:

Cohort A: Subjects presenting for screening mammograms and subsequently confirmed to NOT have a breast malignancy by radiologic exam and did not have any prior malignancy excluding non-melanotic skin cancers or cancers for which they had been treated and remained in remission for greater than or equal to 5 years.

Cohort B: Subjects presenting with a new breast cancer that had not metastasized and the primary treatment modality was surgery. Patients were excluded from this group if they had stage IV breast cancer.

Cohort C: Subjects with metastatic or recurrent/relapsed breast cancer.

**Figure 1:**
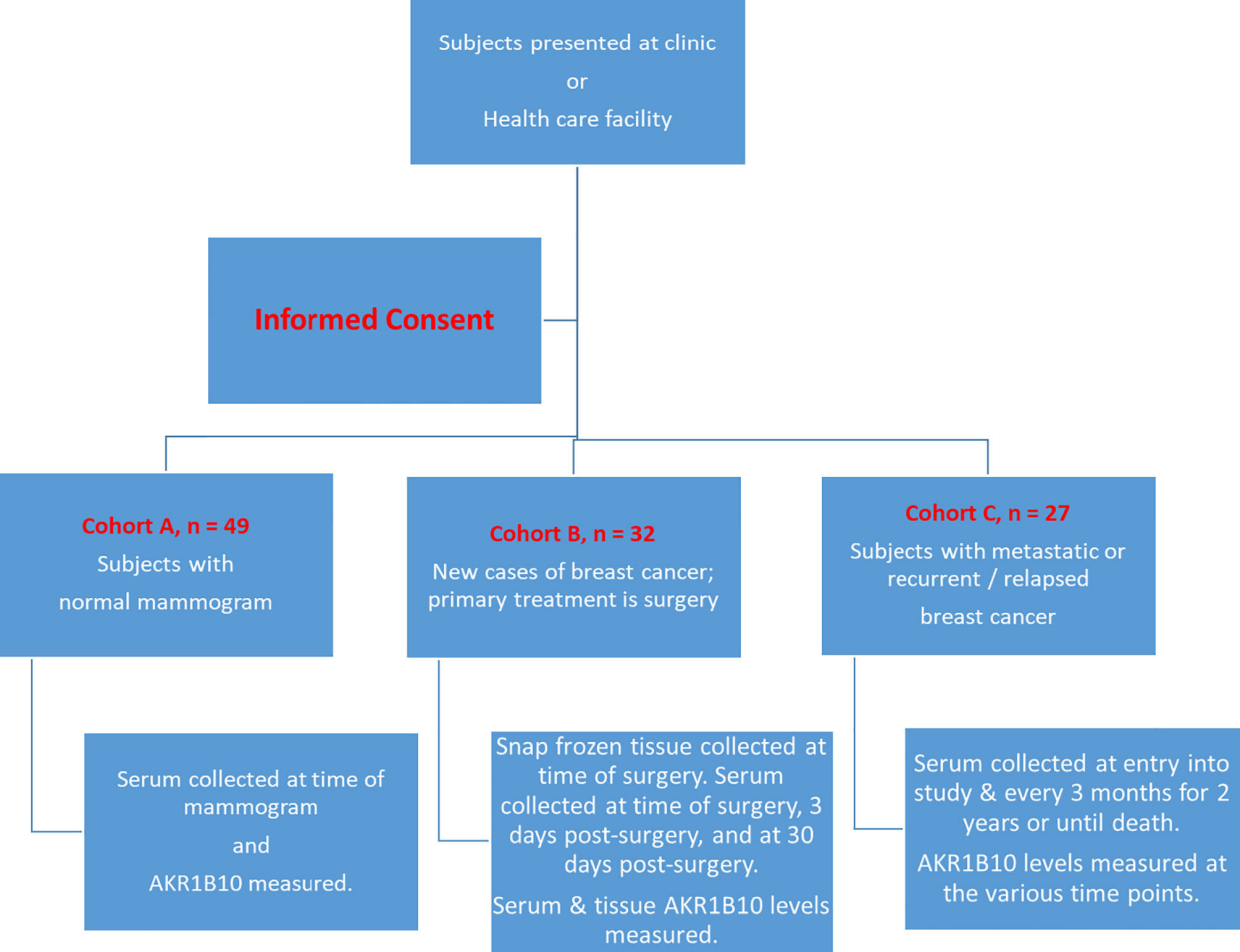
Flow Chart of Schema of the Study. Subjects fit one of the three cohorts and were then followed and tested as per the protocol outlined.

A flow chart with the various cohorts and treatments offered is illustrated in **Figure 1**.

### Serum Sample Collections

A standard operating procedure for collections of serum specimens used. In brief, approximately 10 mL of whole blood was collected in BD Vacutainer red top glass collection tubes (lot # 366430), provided by the hospital or SCI, with no additives using a standard vacutainer. The blood was allowed to sit for 15-30 minutes in order to clot, after which the tubes were centrifuged at 1890 g (2850 rpm) for 15 minutes at room temperature. Serum was immediately transferred into clean polypropylene tubes (100 µL per tube) using an Eppendorf pipette, encoded with a number, and stored at ‐80°C. Serum samples that were improperly processed were excluded from study use.

### Serum AKR1B10 Measurement

AKR1B10 protein in serum was measured by a sandwich enzyme‐linked immunosorbent assay (ELISA) made in house (29). In brief, a polyclonal antibody from a goat against the whole AKR1B10 protein was used as a capture antibody, and a rabbit antibody against an AKR1B10-specific peptide was used as a detection antibody which was well characterized (18). The sandwich ELISA was conducted as follows: High-binding 96-well plates were coated with 100μl of 7μg/ml capture antibody and incubated at 4°C overnight. After wash three times with PBS, the wells were blocked with 250μl of blocking buffer (Alpha Diagnostic, TX) at 37°C for 2 hours. Samples (100μl each) were added into the wells in duplicates. Plates were incubated at 37°C for 1 hour, washed five times with PBST (PBS containing 0.05% Tween 20), and incubated at 37°C for 1 hour with 100μl per well of biotin-labelled detection antibody diluted at 1:500 with antibody diluent (Alpha Diagnostic, TX). After being washed five times with PBST, plates were incubated at 37°C for 30 min with 100μl per well of streptavidin–HRP (horseradish peroxidase) conjugates diluted at 1:5000 with antibody diluent. Specific binding was detected at 37°C for 20 min with 100 μl of TMB (HRP Substrate; Thermo Scientific, FL). Reactions were stopped with 50 μl of stop solution (Alpha Diagnostic, TX) and A450 values were read within 30 min with A620nm as a reference. Purified recombinant AKR1B10 protein was used for a standard curve for concentration calculation. PBS was used as blank and mouse serum was used as a negative control.

### Western Blot

Tissues were homogenized in a cell lysis buffer (Roche, IN) containing a cocktail of protease inhibitors and soluble proteins were collected at 20, 800 g (14,000 rpm) for 15 min. Protein separation, blotting and antibody detection were conducted as described previously (20). β‐Actin was used as a control of protein loaded. The antibody used for detection of AKR1B10 protein was made in house and well characterized. This antibody is highly specific to AKR1B10 protein. Please see the **Supplementary Data** in reference (18) for details.

### RNA Preparation and RT–PCR

Total RNA was extracted from frozen tissues using a Trizol^®^ reagent (Invitrogen, CA). For semi-quantitative RT‐PCR, RNA (1.0μg) was de-contaminated of DNA with RNase‐free DNase 1 (Invitrogen, CA), followed by the first‐strand cDNA synthesis with oligo‐dT primers and Superscript II^®^ retrotranscriptase (Invitrogen, CA). PCR was run with the Platinum PCR SuperMix following the protocol (Invitrogen, CA). β‐Actin mRNA in each sample was used as an internal control. Primers for AKR1B10 were 5′‐CTG GAT CCG GCA AGA TTA AGG AGA T (forward) and 5′‐GAC TGC GGC CGC GAT ATC CAC CAG G (reverse) and for β‐actin primers were 5′‐ATC ATT GCT CCT CCT GAG CGC (forward) and 5′‐TGA ACT TTG GGG GAT GCT CGC (reverse).

### Immunohistochemistry

Paraffin-embedded tissue sections were de-paraffined and hydrated, and antigens were retrieved with standard procedures. After being blocked with 5% horse serum for 30 min, sections were incubated with a specific AKR1B10 antibody as previously described (20). AKR1B10 expression was evaluated blindly by at least a researcher and a pathologist and scored by staining intensities from “0” to “3”, representing no (“0”), low (“1”), intermediate (“2”) or high expression (“3”), respectively. The antibody used for detection of AKR1B10 protein was made in house and well characterized. This antibody is highly specific to AKR1B10 protein. Please see the references (18, 29, 32) for details.

### Statistical Analysis

Descriptive statistics were examined for all variables. Spearman rank correlation coefficients were used to assess the relationship between AKR1B10 expression and continuous or ordinal variables or serum AKR1B10 levels. Wilcoxon rank‐sum tests or Kruskal–Wallis tests were used with categorical variables. Kaplan–Meier plots were produced to examine the relationship between AKR1B10 expression and patient survival and log‐rank test was used for statistical tests. Results were considered statistically significant at p < 0.05.

## Results

### Subject/Patient Characteristics

**Table 1** summarizes the characteristics of subjects/patients. Cohort A (n=49) included women >18 years old with normal mammography, i.e., Breast Imaging Reporting and Data System score of 1 or 2 (BIRADS 1 or 2). The subjects were 91.8% Caucasian, 6.2% African American, and 2.0% Asian. Co-morbidities in Cohort A included acute maxillary sinusitis (2%), allergic rhinitis (10%), anxiety (33%), dysrhythmias (10%), arthralgias/arthritis (12%), asthma (14%), atrial fibrillation (2%), atrial flutter (2%), Bell’s palsy (2%), bursitis (2%), chondrodermatitis nodularis helicis (2%), chronic bronchitis (2%), chronic kidney disease (2%), chronic serous otitis media (2%), congestive heart failure (4%), COPD (4%), coronary artery disease (12%), DVT/PE (2%), depression (30%), dermatitis (2%), detached retina (2%), diabetes mellitus (16%), diverticulosis (4%), endometriosis (2%), epilepsy (2%), eczema (4%), fibromyalgia (2%), GERD (16%), gout (4%), Grave’s disease (2%), hematochezia (4%), remote history of Hodgkin’s lymphoma (2%), homonymous hemianopsia (2%), hypertension (44%), hypothyroidism (14%), ileitis (2%), intracranial hypertension (2%), irritable bowel syndrome (4%), keratoconjunctivitis sicca (2%), migraine (10%), myocardial infarction (2%), obesity (14%), onchmycosis (2%), osteopenia (8%), osteoporosis (2%), Parkinson’s disease (2%), periodic limb movement disorder(2%), pituitary disorder (2%), positional vertigo (2%), Raynaud’s phenomenon (2%), restless leg syndrome (2%), rheumatoid arthritis (2%), sleep apnea (4%), OSA (12%), TIA (2%), ulcerative proctosigmoiditis (2%), vascular disease (2%), and ventral hernia (2%). Cohort B (n=32) included newly diagnosed breast cancer patients ranging in stages from Stage 0 to Stage III, with an average tumor size of 1.966 cm. The average age of the patients was 60.5 years with a mean body mass index (BMI) of 33. The patients were 90.6% Caucasian, 6.3% African American, and 3.1% Asian. Percentage of patients with ER positivity was 81.2%, PR positivity was 71.9%, and HER-2 positivity was 7.4%. Histologic grade was grade 1 (19.4%), grade 2 (53.1%), and grade 3 (29.0%). Co-morbidities for Cohort B included acute kidney injury (3%), allergic rhinitis (22%), anemia (3%), anxiety (9%), anxiety (30%), dysrhythmia (9%), arthritis (6%), asthma (6%), atrial fibrillation (9%), bipolar disorder (3%), bursitis (3%), oral candidiasis (6%), cellulitis (3%), CVA 93%), history of cervical cancer (3%), history of chest pain (3%), cholelithiasis (6%), chronic kidney disease (3%), COPD (6%), history of colon cancer (3%), congestive heart failure (6%), coronary artery disease (15%), DVT (9%), depression (45%), diabetes mellitus (18%), diverticulosis (3%), emphysema (3%), eczema (3%), fibromyalgia (3%), GERD (18%), gout (3%), prior HSV infection (3%), hydraadenitis (3%), hydronephrosis (3%), hypertension (63%), hyperthyroidism (3%), hypothyroidism (21%), irritable bowel syndrome (3%), nephrolithiasis (3%), obesity (21%), OSA (3%), obstructive pulmonary syndrome (3%), onchomycosis (3%), osteoarthritis (15%), osteopenia (12%), osteoporosis (6%), panic disorder (3%), parotitis (3%), peripheral vascular disease (3%), pharyngitis (3%), past pneumonia (12%), polycystic ovaries (3%), post traumatic seizure (3%), pre-diabetes (3%), PTSD (3%), pulmonary embolism (9%), pyelocystitis (3%), restless leg syndrome (6%), prior sinusitis (3%), tachycardia (3%), tendinitis (3%), and ventral hernia (3%). Tumors were categorized as luminal A (70.4%) luminal B (7.4%), and basal (22.2%). Oncotype Dx and Mammaprint scores were available for some of the patients (**Table 1**). Treatment was lumpectomy or mastectomy. In Cohort C (n=27), all patients had metastatic diseases with following sites: bones, liver, lung or others. The average age of patients was 55.8 years, and mean BMI was 33.4. The patients were 92.5% Caucasian and 7.5% African American. The percentage of patients with ER positivity was 81.5%, PR positivity was 46.2%, and HER-2 positivity was 16.0%. Histologic grade of tumors was 44.4% in grade 2 and 40.7% in grade 3. Tumor subtypes were 66.7% luminal A, 7.4% luminal B, 7.4% HER-2, and 11.1% basal.

**Table 1:** Patient characteristics.

	Cohort A (Healthy Women, n = 49)	Cohort B (Localized, n = 32)	Cohort C (Metastatic, n = 27)
Mean Age	64.6 years	60.5 years	55.8 years
Gender	All female		
Male		0 %	3 (11%)
Female		32 (100 %)	24 (89%)
Mean body mass index (BMI)	N/A	33	33.4
Races			
Caucasians	45 (91.8%)	29 (90.6%)	25 (92.5%)
American Africa	3 (6.2)	2 (6.3 %)	2 (7.5%)
Asian	1 (2.0)	1 (3.1%)	0
Mean Tumor size (cm)	NA	1.966	Metastatic disease
Histologic grade	NA		
Grade I		6 (19.4 %)	0
Grade Ii		17 (53.1%)	12 (44.4%)
Grade Iii		9 (29%)	11 (40.7%)
Missing		0	4 (14.81%)
Tumor invasiveness	NA		
Invasive		27 (84.4%)	All metastatic or recurrent.
Non-Invasive		5 (15.6%)	
HR status	NA		
ER +ve		26 (82 %)	22 (81.5%)
ER –ve		6 (18.8 %)	5 (18.5%)
PR +ve		23 (71.9%)	12 (46.2)
PR –ve		8 (25%)	15 (55.5 %)
HER-2 +ve		2 (7.4%)	4 (16%)
HER-2 –ve		25 (92.6%)	21 (84%)
Molecular subtypes	NA		
Luminal A		19 (70.4 %)	18 (72 %)
Luminal B		2 (7.4%)	2 (8 %)
Basal		6 (22.2%)	3 (12 %)
HER-2 expressing		--	2 (8 %)
Missing		5 (15.6%)	2 (8%)
Oncotype Dx	NA	>25 = 1	N/A
(available 13/32)		<25 = 12	

N/A or NA, not applicable.

### Serum AKR1B10 Is Increased in Breast Cancer Patients and Correlated With Expression in Tissues

**Figure 2:**
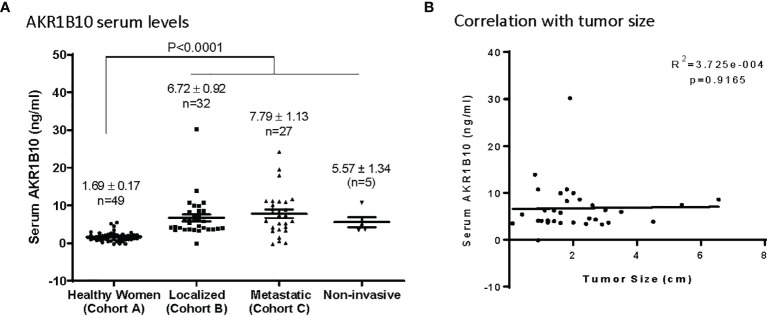
Serum levels of AKRlBlO in Breast Cancer patients. **(A)** AKR1B10 serum levels in healthy women and patients with localized, metastatic, or noninvasive disease. Serum AKR1B10 levels were significantly higher in breast cancer patients than that in healthy women. **(B)** Serum AKR1B10 levels in localized breast cancer patients failed to show any direct correlation with the tumor size.

Serum levels of AKR1B10 were measured in subjects enrolled and, as noted in **Figure 2A**, were significantly elevated in patients with localized (6.72 ± 0.92 ng/ml) or metastatic (7.79 ± 1.13 ng/ml) disease as opposed to the cohort of cancer-free healthy women who had a mean serum value of 1.69 ± 0.17 ng/ml. However, in cohort B of breast cancer patients (localized), the serum AKR1B10 levels were not notably correlated with the size of tumors (**Figure 2B**).

**Figure 3:**
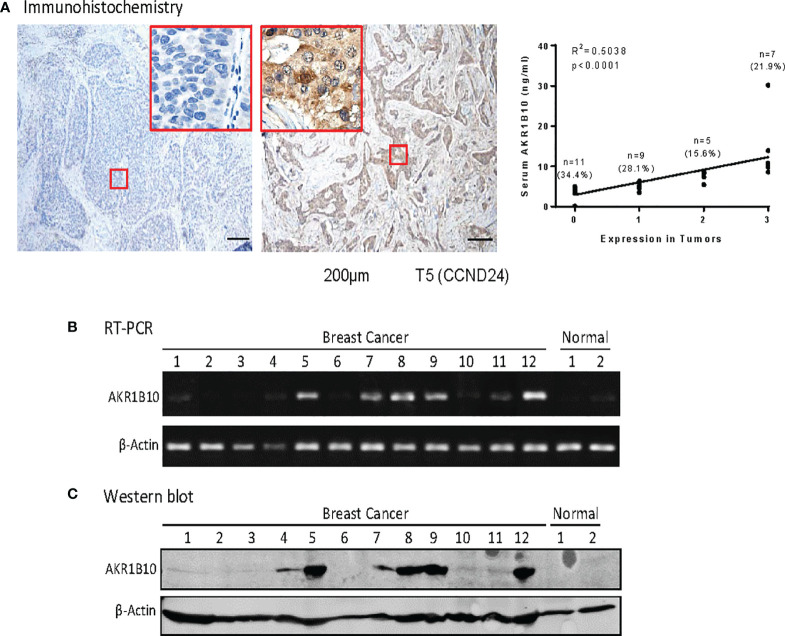
AKR1810 expression in breast cancer tissues. **(A)** Immunohistochemistry. Paraffin embedded tissue sections were used for Immunohistochemical staining of AKR1B10. The serum levels of AKR1B10 were significantly correlated with its expression in tumor tissues. **(B)** Semi­ quantitative RT-PCR for AKR1B10 mRNA and **(C)** Western blot for AKR1B10 protein. Fresh tissues were collected from some cases to run RT-PCR and Western blot to confirm AKR1B10 expression. As compared to tumor specimens, healthy tissues showed very weak expression of AKR1B10 mRNA by PCR **(B)** and undetectable AKR1B10 protein by Western blot. Breast cancer tissues Line 1to 12: CAND4, CAND8, CAND21, CAND22, CAND24, CAND25, CAND29, CAND33, CAND34, CAND36, CBMR28, CCN4 (a case recruited for neoadjuvant trial); Normal breast tissues Line 1 & 2: normal breast tissues from tissue bank.

Expression of AKR1B10 was further evaluated in cancer tissues with immunohistochemistry (IHC). Our data exhibited that AKR1B10 was highly expressed (scored at 3) in 7 (21.9%) of 32 breast cancer tissues (**Figure 3A**), moderately expressed (scored at 2) in 5 (15.6%) cases, and expressed at a low level (scored at 1) in 9 (28.1%) cases. AKR1B10 was not detected (scored at 0) in 11 (34.4%) cases. The serum value of AKR1B10 was correlated with its expression in tumor tissues (**Figure 3A**, *right*). AKR1B10 expression in cancer tissues was confirmed by RT-PCR and Western blot (**Figures 3B, C**; **Supplementary Figure 1**). AKR1B10 was also studied in a group of patients presenting with large tumors who underwent neoadjuvant chemotherapy, and data from one sample (CCN4) is presented. These findings will be discussed in a separate publication. RT-PCR demonstrated very week expression of AKR1B10 mRNA in normal breast tissues, but Western blot did not detect AKR1B10 protein.

### Serum AKR1B10 Is Rapidly Decreased After Surgical Removal of Primary Tumors

**Figure 4:**
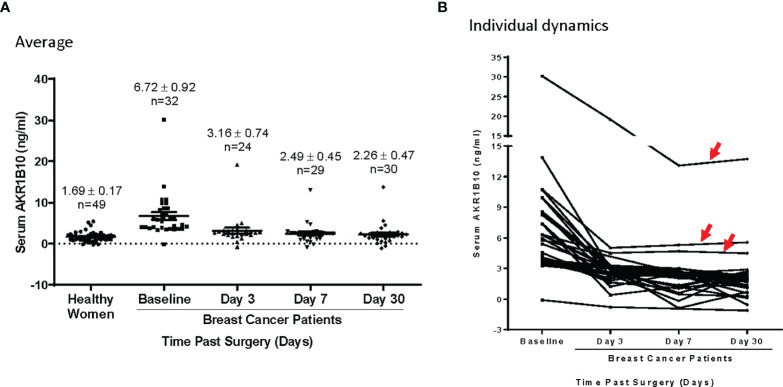
**(A)** AKR1B10 level dynamics in patients undergoing lumpectomy or mastectomy. The AKR1B10 levels significantly dropped on day 3 and plateaued thereafter till day 30 to baseline similar to healthy controls. **(B)** In this collection, 3 cases (red arrows) showed dramatic drops of serum AKR1B10 post surgery but maintained a level that was higher than healthy control. This indicates that these case continued expression of AKR1B10 probably secondary to micro metastasis.

We monitored the serum AKR1B10 levels in patients who had localized breast cancer and underwent lumpectomy or mastectomy. As shown in **Figure 4**, the serum AKR1B10 returned to the basal level within 3 days after surgical removal of primary tumors except for 3 cases, suggesting that the half-life is less than 3 days. In most patients, the serum AKR1B10 level plateaued beyond day 3 and achieved a new baseline similar to that of healthy women, indicating the specificity of serum AKR1B10 to primary breast tumors. However, it was noted that 3 (9.4%) out of 32 patients continued to produce a quantity of AKR1B10 protein above the surmised basal level. In these patients, no obvious sources or secondary non-malignant conditions were identified to be associated with excessive AKR1B10 production. A putative explanation may be the presence of micro-metastasis not detected by instrument examination in clinic.

### Serum AKR1B10 Is a Potential Diagnostic Marker

**Figure 5:**
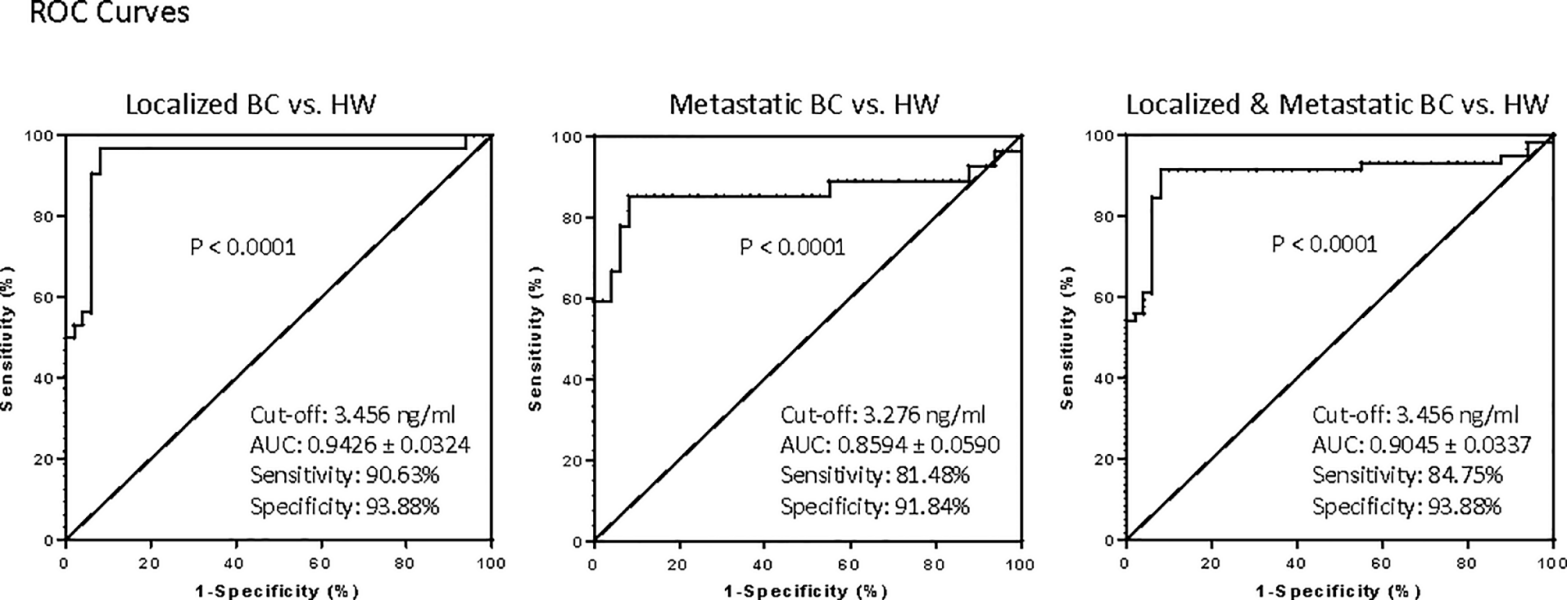
Receiver operating characteristic (ROC) curves for AKR1B10 levels for diagnostic interpretation based on Youden's index (sensitivity plus specificity -1).The ROC curves were plotted for localized, metastatic and combined localized and metastatic breast cancer patients against healthy women. The optimal diagnostic value of AKR1B10 in our data set of combined localized and metastatic breast cancer patients was 3.456ng/ml,with a specificity of 93.88% and a sensitivity of 84.75%,suggesting its potential diagnostic value.

We further estimated the potential diagnostic value of the serum AKR1B10 in breast cancer. We plotted ROC curves for serum AKR1B10 with breast cancer versus healthy women (**Figure 5**), and calculated optimal cut-offs based on Youden’s Index, i.e., sensitivity plus specificity minus one, which indicates the capability of detecting outbreaks and non-outbreaks. When the ROC curve for AKR1B10 was plotted with the cohort of localized breast cancer (n = 32) versus healthy women (n = 49), optimal diagnostic cut-off value of serum AKR1B10 was at 3.456 ng/mL, which yielded AUC 0.9426 ± 0.0324 (95% CI 0.8790 - 1.006; p < 0.0001), sensitivity 90.63% (95% CI 74.98% to 98.02%), and specificity 93.88% (95% CI 83.13% to 98.72%). When the ROC curve for AKR1B10 was plotted with the cohort of metastatic breast cancer (n = 27) versus healthy women (n = 49), optimal diagnostic cut-off value of serum AKR1B10 was at 3.276 ng/mL, which yielded AUC 0.8594 ± 0.0590 (95% CI 0.7437 – 0.9705; p < 0.0001), sensitivity 81.48% (95% CI 61.92% to 93.70%), and specificity 91.87% (95% CI 80.40% to 97.37%). When the ROC curve for AKR1B10 was plotted with the cohorts of localized plus metastatic breast cancer (n = 59) versus healthy women (n = 49), optimal diagnostic cut-off value of serum AKR1B10 was at 3.456 ng/mL, which yielded AUC 0.9045 ± 0.0337 (95% CI 0.8384 – 0.9706; p < 0.0001), sensitivity 84.75% (95% CI 73.01% to 92.78%), and specificity 93.88% (95% CI 83.13% to 98.72%). These data indicate the potential values of serum AKR1B10 as a diagnostic marker of breast cancer.

### AKR1B10 Is Expressed in DCIS Tissues and Elevated in Serum of Patients With Non-Invasive Breast Cancer

**Figure 6:**
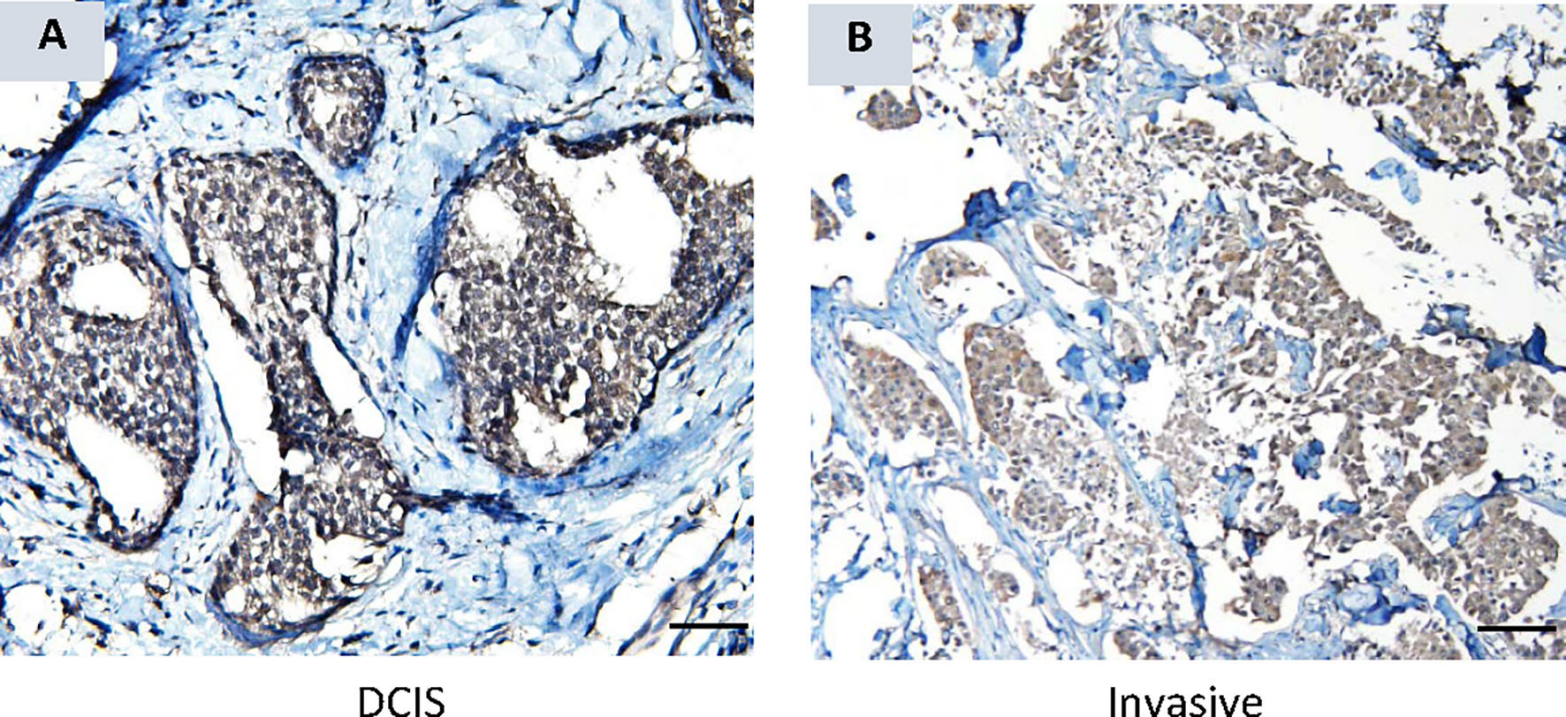
AKR1B10 expression by immunohistochemistry in ductal carcinoma in situ (DCIS). **(A)** AKR1B10 expression in DCIS, and **(B)** AKR1B10 expression in invasive cancer for a comparison.

Cohort B, which consisted of localized breast cancer, included 5 cases of diagnosed DCIS. The serum AKR1B10 of these 5 cases was at 5.57 ± 1.34 ng/ml, significantly higher than that in healthy women with normal mammograms (1.69 ± 0.17 ng/ml, p<0.0001) (**Figure 2**). More interestingly, 3 (60.0%) of 5 DCIS cases had a serum AKR1B10 level higher than the cut-off of 3.456 ng/ml. IHC data confirmed the expression of AKR1B10 in DCIS tissues (**Figure 6**; **Supplementary Figure 2**). These data indicate the potential of serum AKR1B10 as a marker of DCIS. It is highly interesting to expand the evaluation of serum AKR1B10 as a marker of DCIS to guide clinical management of this early disease as overtreatment of DCIS is a sophisticated clinical issue.

### Serum AKR1B10 Levels Do Not Correlate With Disease Burden, Oncotype Dx Scores, Molecular Subtypes, or Histological Stages

**Figure 7:**
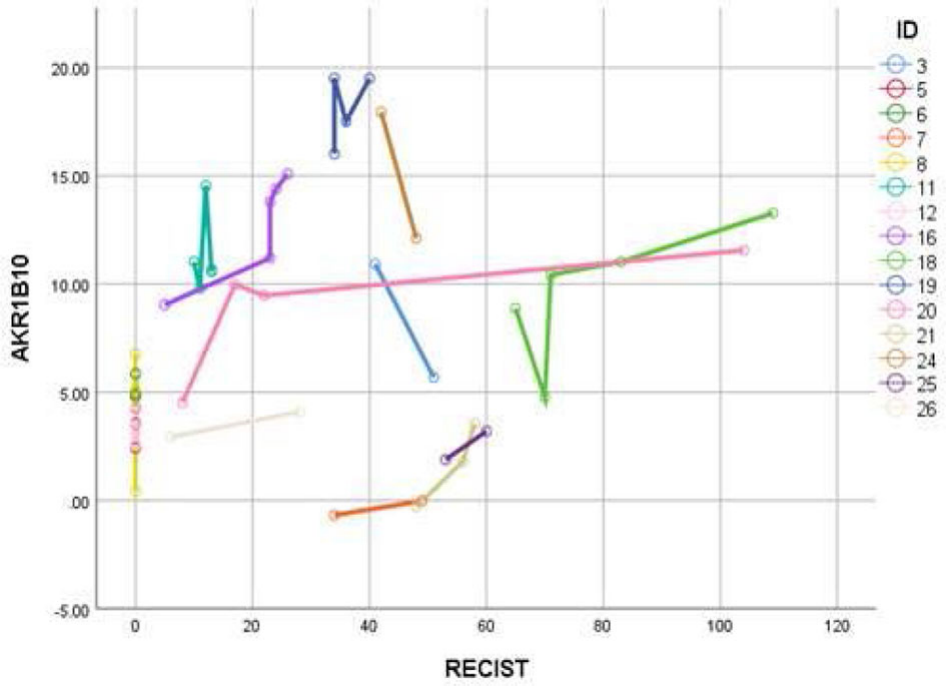
AKR1B10 levels did not show any correlation with RECIST scores. Higher RECIST scores suggest high disease burden and vice versa. Data suggest that serum AKR1B10 may not be used for monitoring disease activity or response.

Data in **Figure 2** demonstrates that AKR1B10 in the serum of localized breast cancer patients was significantly elevated, but there was no correlation between tumor size and the level of serum AKR1B10. Likewise, in the metastatic settings, serum levels were elevated, and the levels fluctuated independently of disease burden. Graphing tumor burden calculated by periodic imaging RECIST criteria) against AKR1B10 serum levels showed no correlation (**Figure 7**). AKR1B10 levels in serum also did not correlate with Oncotype Dx scores (**Table 2**). The scores in our cohort of localized breast cancer patients ranged from 2 to 29, spanning the range from low risk to high risk disease (data not shown). Similarly, the serum AKR1B10 levels were not correlated with ER, PR and HER-2 status, molecular subtypes, or histological grades (**Table 2**).

**Table 2:** Correlation between AKR1B10 levels and hormonal receptor status, oncotype DX scores, and molecular subtypes.

	Cohort B (Localized, n = 32)		Cohort C (Metastatic, n = 27)	
	AKR1B10 (ng/ml)	P values	AKR1B10 (ng/ml)	P values
ER status		0.944		0.928
ER +	6.26 ± 3.10		7.80 ± 6.22	
ER -	9.27 ± 10.33		6.38 ± 4.49	
PR Status		0.707		0.035
PR +	6.17 ± 3.14		10.30 ± 7.05	
PR -	8.97 ± 8.86		5.45 ± 3.78	
HER-2 status		0.205		0.108
HER-2	9.06 ± 2.39		3.55 ± 2.52	
HER-2 -	6.85 ± 5.65		8.77 ± 6.05	
Onc Dx Scores		N/A		N/A
Onc Dx < 25	>25 = 1		N/A	
Onc Dx > 25	<25 = 12			
Molecular subtypes		N/A		N/A
Luminal A	6.09 ± 3.21		8.69 ± 6.52	
Luminal B	9.06 ± 2.39		5.08 ± 1.09	
Basal	9.27 ± 10.33		9.28 ± 2.10	
HER-2 expressing	NA		2.03 ± 2.92	
Histologic grade				0.634
Grade I	4.82 ± 2.54	0.209	NA	
Grade II	6.42 ± 3.17		9.26 ± 7.97	
Grade III	8.43 ± 8.45		7.66 ± 5.79	

N/A or NA, not applicable.

## Discussion

MUC1 is a large transmembrane glycoprotein which is frequently overexpressed and aberrantly glycosylated in breast cancer. CA 15-3 and CA 27.29 antigens are from the same gene—MUC1—which codes for a mucin glycoprotein. Mucin glycoprotein (also known as polymorphic epithelial mucin, PEM) is found on ductal cell surfaces and most glandular epithelia. Physiologically, these glycoproteins function in cell protection and lubrication (33). In certain malignancies (e.g., kidney, liver, and breast), these glycoproteins can shed into the circulation due to disruption of cell polarity (33). As a result, elevated CA 15-3 and CA 27.29 serum concentrations have been correlated with cancer growth and/or progression. Although CA15-3 and CA27-29 are frequently elevated at the time of initial diagnosis and vary with the bulk of disease present, their levels are influenced by a variety of benign conditions, and the levels are not sensitive enough to correctly make the initial diagnosis. CA15-3, CA125, and CEA, even when combined with tumor-associated autoantibodies, were able to achieve 81.0% sensitivity and 78.8% specificity for detection of breast cancer at best with an area under the receiver operating characteristic (ROC) curve (AUC) of 0.89 (34). ASCO guidelines currently do not recommend routine use of these tumor markers. As such, these markers have predominantly been relegated to research roles without significant clinical utility.

Additionally, a number of other serum and tissue tumor markers have been examined in breast cancer. The role of estrogen receptor (ER), progesterone (PR), and HER-2 are well established (35). High levels of uPA, PAI-1, and uPAR in tumor tissues have correlated with poor prognosis in several types of human cancers, including breast, endometrial, ovarian, colon, lung, stomach, and renal cancer (36). Cathepsin D is a protease that aids in the digestion of exhausted and/or denatured cell proteins. Cathespsin D is secreted into the serum of breast cancer patients, and its overexpression has been correlated with poorer prognosis (37). Elevated levels of cyclin E have also been associated with a poorer prognosis (38). Nestin, an intermediate filament, is often present in substantial quantities in basal breast cancer and may be a marker for highly aggressive disease (39). Finally, HE4 has been reported to be elevated in breast cancer patients and has also been proposed as a biomarker (40).

Our data suggest a clear distinction of serum AKR1B10 between the subjects with no evidence of breast malignancy versus those with either pre-invasive or invasive breast diseases, including metastatic disease. ROC plotting analyses demonstrated that the serum AKR1B10 was a promising diagnostic marker with high sensitivity and specificity in both localized and metastatic diseases. Very interestingly, even the patients with pre-invasive disease displayed an elevated level of the protein, suggesting its use as a harbinger of breast pathology. There is a paucity of data on the use of either CA15.3 or CA27.29 in the diagnosis of DCIS/LCIS, and our data indicates that AKR1B10 is elevated in these premalignant conditions, being a potential marker for clinical management of the premalignant breast diseases. This study also conducted a dynamic observation of serum AKR1B10 before and after surgery, and our data showed that the serum AKR1B10 rapidly declined in most patients within 3 days after surgical removal of the primary tumor. This indicates a very specific relationship between serum AKR1B10 levels to the primary breast tumors. However, it is noteworthy that in three cases, the serum AKR1B10 did greatly decline after removal of the primary tumor, but plateaued at a high level after three days. Clinical data of these three cases did not support the presence of other malignancies or other conditions which may increase the serum AKR1B10 levels. Perhaps these cases had micro-metastatic disease that were not detected by clinical diagnostic tools. If true, the serum AKR1B10 may be a promising marker to identify early metastasis of breast cancer, and further study is warranted.

We analyzed the correlation of serum AKR1B10 with its expression in tumor tissues and tumor burden. We realized that the serum level of AKR1B10 was correlated to its expression in tumor tissues, but not to the tumor size. In metastatic diseases, the serum AKR1B10 levels were not correlated to the RECIST value either. Therefore, our study results do not support the serum AKR1B10 as a marker of the disease burden in metastatic diseases. It is noteworthy that in these cohorts, the expression of AKR1B10 evaluated by RT-PCR and Western blot was in a relatively low number of tumor tissues compared to the previous reported (20). We do not have a direct explanation at moment, but it is possible that the relative low percentage of AKR1B10 expression in breast cancer tissues may be derived from individual differences as subjects investigated in these two separated studies were clearly different populations of patients. The whole membranes for the Western blot are included as **Supplementary Figure 1** in the revised manuscript for reference.

AKR1B10 also appeared to be elevated in all subtypes of breast cancer. This feature is consistent with other breast cancer markers, such as CEA, CA15.3, and CA 27.29. Yerushalmi, et al. (41) studied 810 patients and noted that luminal subtypes of breast cancer were associated with at least one elevated tumor marker in nearly 90% of cases, but these tumor markers were less frequently elevated in other subtypes with 74.1% of basal tumors and 71.4% of non-basal triple negative tumors displaying elevation of at least one tumor marker. The location of metastases and the number of sites of diseases did not impact levels. Our findings with AKR1B10 mirror the findings of these past markers.

AKR1B10 expression in normal human tissues is largely confined in the gastrointestinal (GI) tract, where it is significantly expressed in the esophagus, stomach, small intestine, colon, and gallbladder (The Human Protein Atlas, https://www.proteinatlas.org/ENSG00000198074-AKR1B10/tissue) (11).. AKR1B10 expression has also been observed in non-neoplastic diseases, such as atopic dermatitis (42), diabetes (43), and leprosy (44). The function of AKR1B10 is also context dependent. It has been described as a tumor suppressor gene in colon cancer (45) and an oncogene in hepatocellular cancer (46), in gastric cancer (28), and in pancreatic cancer (12). The low expression of AKR1B10 has also been described as an independent prognostic factor in nasopharyngeal cancer (47), hinting a tumor suppressive role in this malignancy. Given its diverse role in several different tumor types and its potential for alterations in benign conditions, utility of AKR1B10 in clinics appears to be the best once a histologic diagnosis of breast cancer is established, and no confounding disease co-exists.

Oncotype Dx assay (Genomic Health Inc., Redwood City, CA) is a clinically validated twenty-one gene assay that stratifies the risk of relapse in breast cancer patients. The test has been used to classify patients as low, intermediate, or high risk. The addition of chemotherapy to hormonal therapy in the high risk group clearly improved survival, while no benefit was obtained for the low risk group, based on analyses of the NSABP B-14 and B-20 trials (48). The TAILORx study (Trial Assigning Individualized Options for Treatment) defined intermediate risk patients as those with scores ranging from 11 to 25 as intermediate and randomized these patients to receive hormonal therapy alone or chemotherapy followed by hormonal therapy. Initial results from the study confirmed good outcomes in low risk patients given hormonal therapy alone. Subsequent results from the study indicated that endocrine therapy was non-inferior to chemoendocrine therapy in terms of local relapse, disease free survival, and overall survival in the intent to treat population (49). However, subgroup analyses indicated that “in women 50 years of age or younger, chemotherapy was associated with a lower rate of distant recurrence than endocrine therapy if the recurrence score was 16 to 20 (percentage-point difference, 0.8 at 5 years and 1.6 at 9 years) or 21 to 25 (percentage-point difference, 3.2 at 5 years and 6.5 at 9 years), although the rates of overall survival were similar”. The net result of the study has been to consider discussing and offering cytotoxic chemotherapy to patients in this category given the trend towards improved disease free survival. Given its ability to differentiate high and low Stage I disease, we think that AKR1B10 may be a protein whose expression in breast cancer tissues can further categorize this intermediate risk group of breast cancer patients and aid the clinician in the decision to administer chemotherapy. This prognostic niche is not unique and has been similarly utilized in other types of malignancies, such as AML, in which molecular markers such as FLT3 and NPM1 risk-stratify patients with normal cytogenetics and normal molecular cytogenetics (50).

In conclusion, this case-controlled pilot trial of AKR1B10 as a serum marker revealed its value as a potential diagnostic marker. Most impressive is the potential of serum AKR1B10 to serve as a serum biomarker of DCIS. Further studies are warranted to assess the clinical application of the serum AKR1B10.

## Data Availability Statement

The original contributions presented in the study are included in the article/**Supplementary Material**. Further inquiries can be directed to the corresponding authors.

## Ethics Statement

The studies involving human participants were reviewed and approved by Springfield Committee for Research Involving Human Subjects (SCRIHS). The patients/participants provided their written informed consent to participate in this study.

## Author Contributions

KrR, ZC, and DC conceived the idea for the study. KD performed statistical analysis. KaR assisted in patient enrollment, data collection, result compilation and statistical analysis. VT assisted with data collection and enrollment. AM contributed in data collection and manuscript review, and XW, YZ, and JG contributed in IHC, Western blot and histology. AH and FZ contributed significantly in manuscript writing, preparation and proof reading. All authors contributed to the article and approved the submitted version.

## Funding

Avon Foundation for Women. The funder was not involved in the study design, collection, analysis, interpretation of data, the writing of this article or the decision to submit it for publication.

## Conflict of Interest

The authors declare that the research was conducted in the absence of any commercial or financial relationships that could be construed as a potential conflict of interest.

## Publisher’s Note

All claims expressed in this article are solely those of the authors and do not necessarily represent those of their affiliated organizations, or those of the publisher, the editors and the reviewers. Any product that may be evaluated in this article, or claim that may be made by its manufacturer, is not guaranteed or endorsed by the publisher.
