# Supplementary material for: AKR1B10 as a Potential Novel Serum Biomarker for Breast Cancer: A Pilot Study
Source: Front Oncol. 2022 Feb 24;12:727505. doi: 10.3389/fonc.2022.727505 (PMC8908957; doi:10.3389/fonc.2022.727505)
Supplement: Supplementary file 1 [file DataSheet_1.pdf]

Supplementary Figure 1

AKR1B10

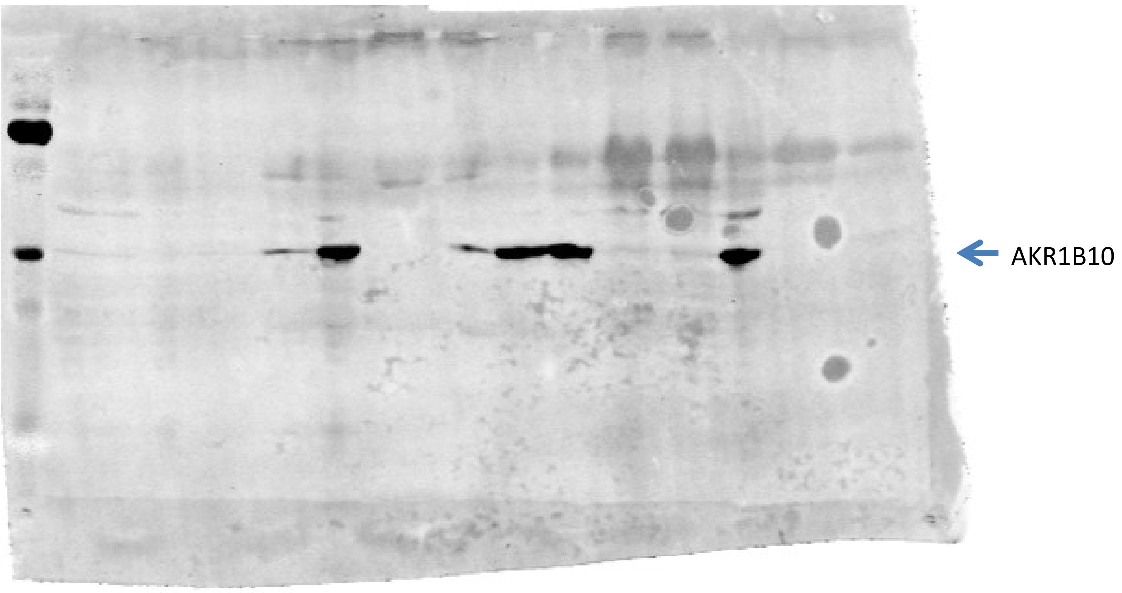

Beta-Actin

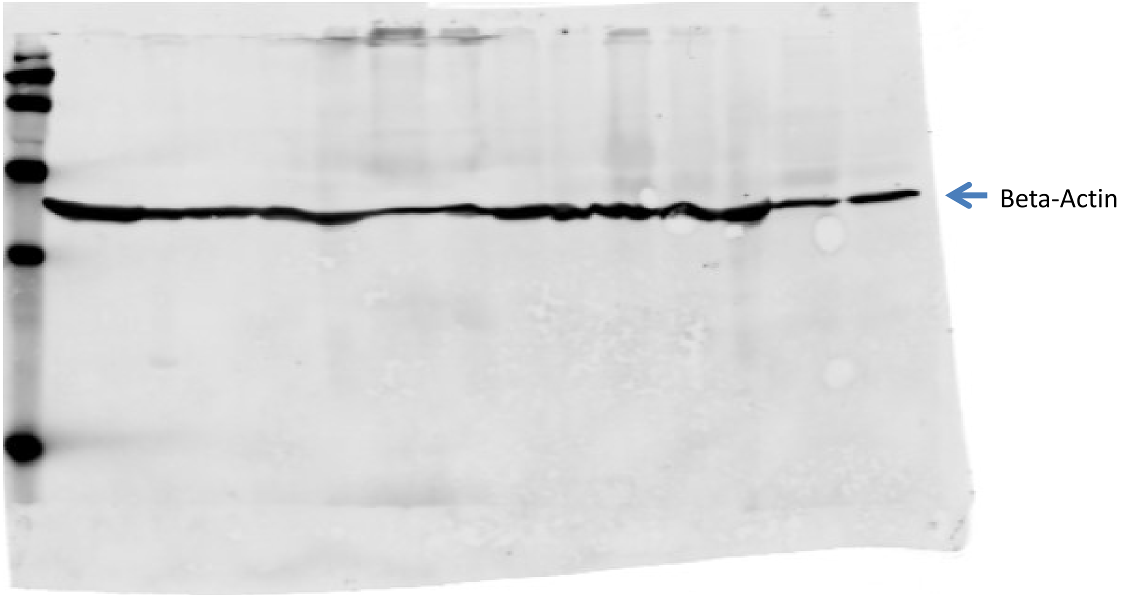

Supplementary Figure 2. Original images of the Western blot.

Supplementary Figure 2

CAND3 (The case presented in Figure 5)

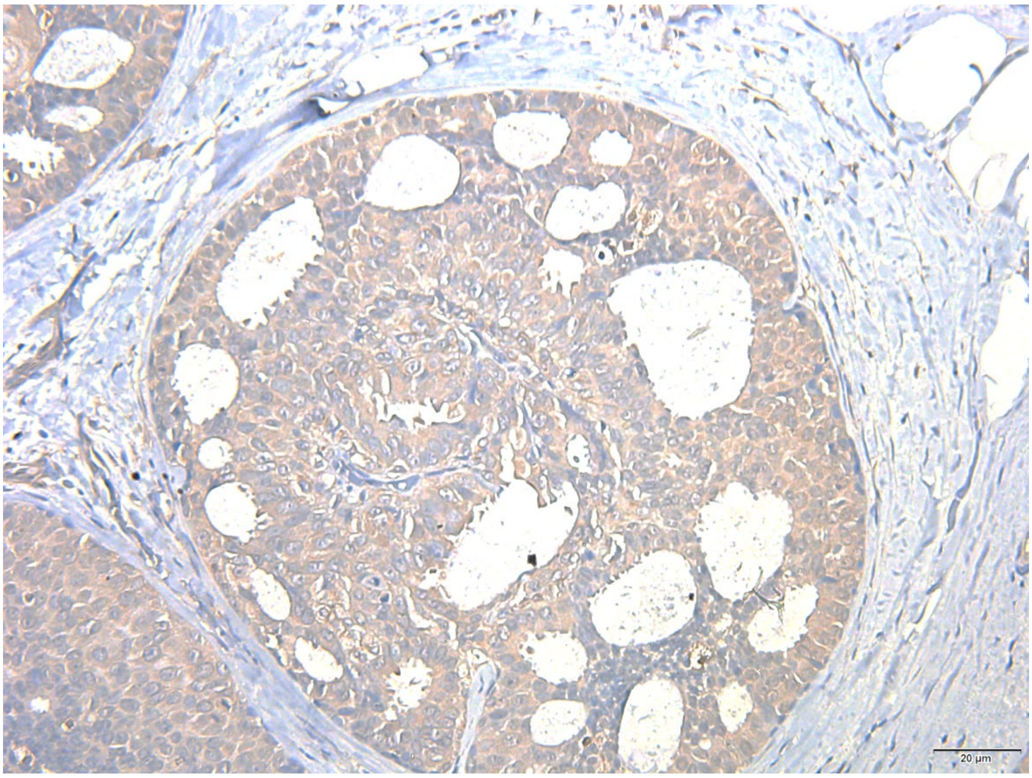

CAND 10

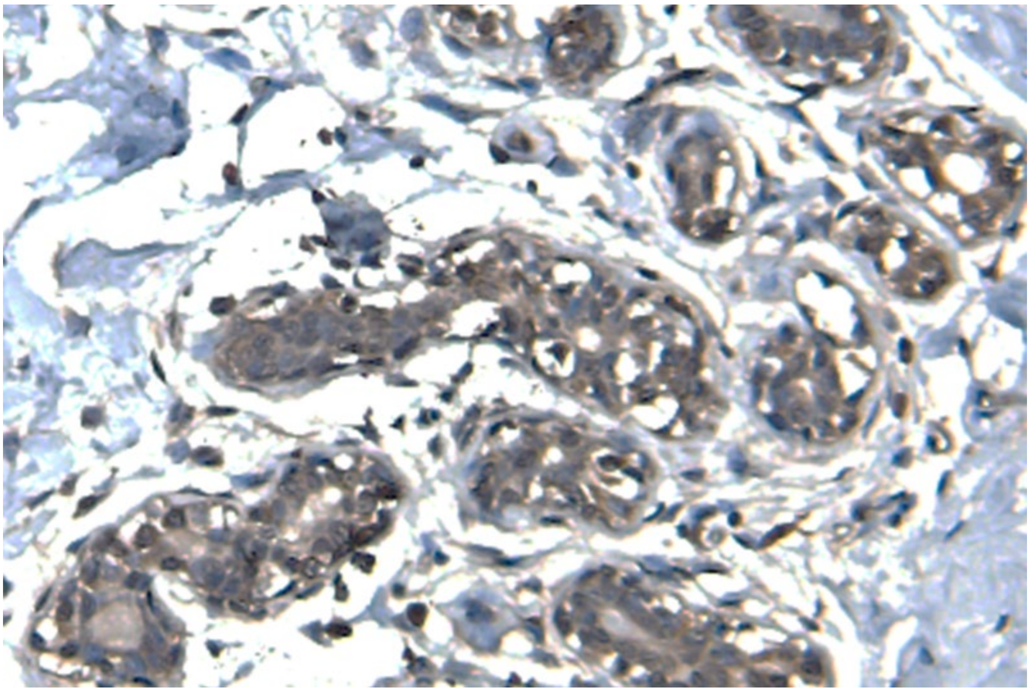

CAND 11

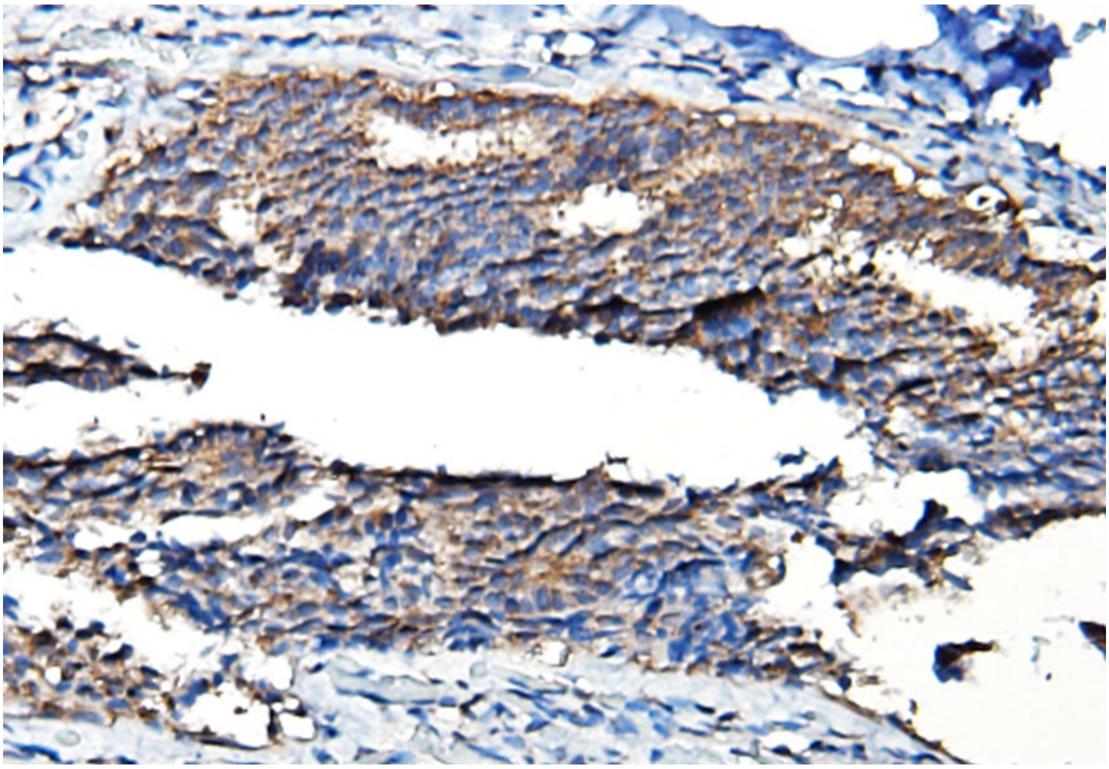

CAND 27

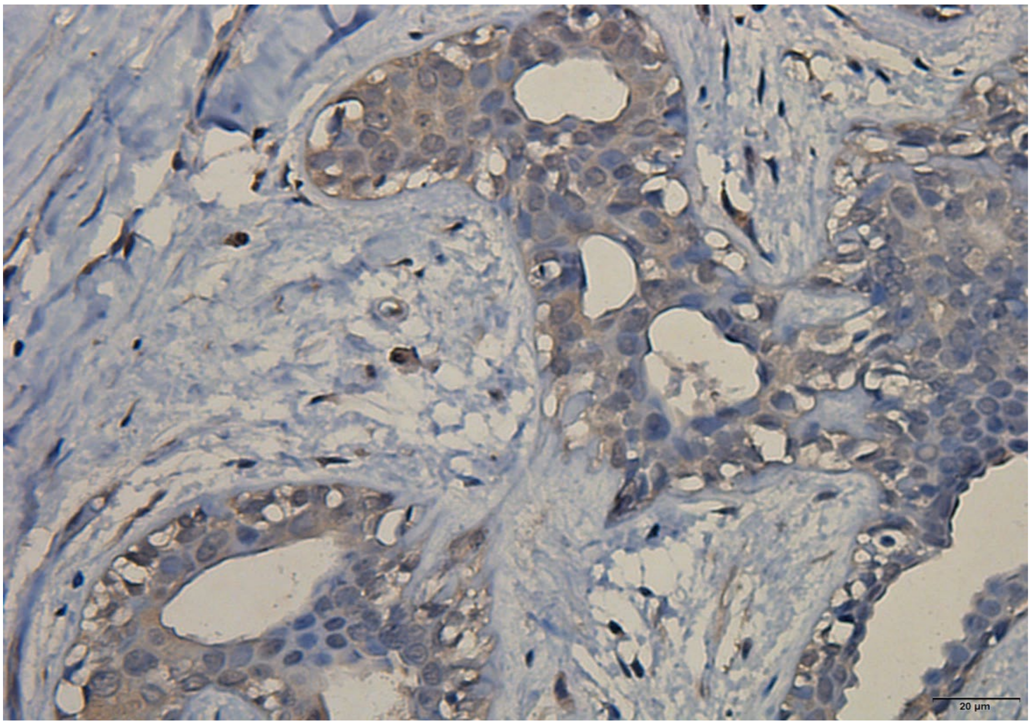

CAND 35

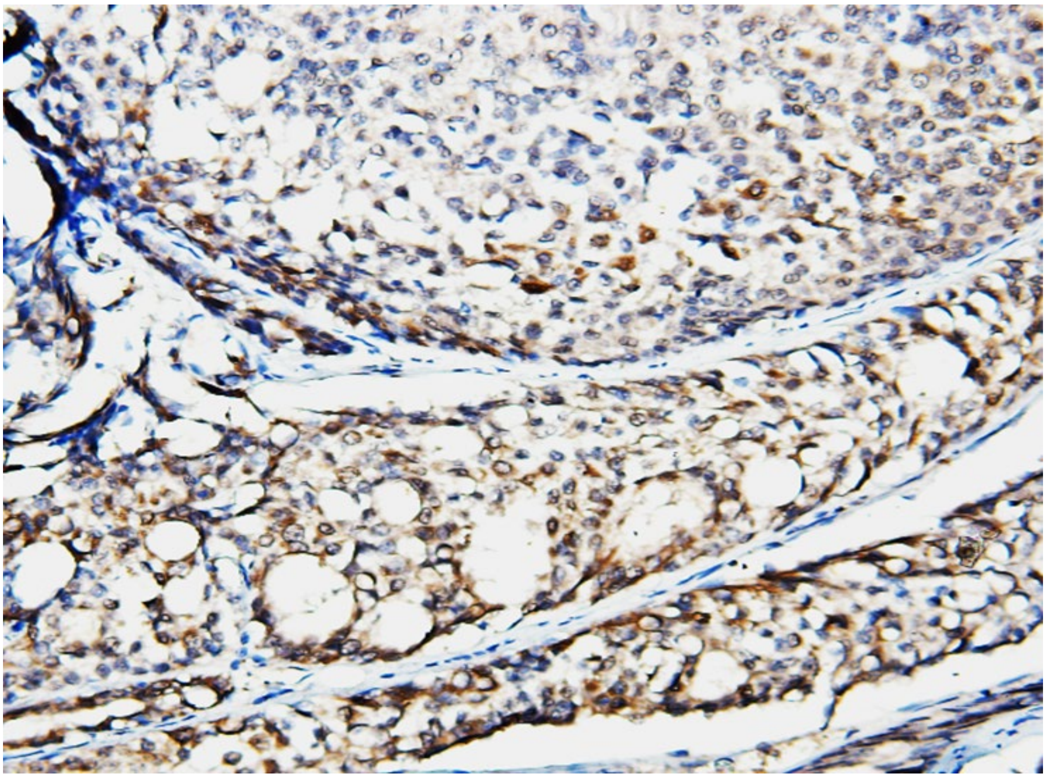

Supplementary Figure 1. IHC images of all 5 cases of DCIS.
