# Supplementary material for: AKR1B10 as a Potential Novel Serum Biomarker for Breast Cancer: A Pilot Study
Source: Front Oncol. 2022 Feb 24;12:727505. doi: 10.3389/fonc.2022.727505 (PMC8908957; doi:10.3389/fonc.2022.727505)
Supplement: Supplementary file 2 [file Image_1.pdf]

ACTIVITY AND SPECIFICITY OF AKR1B10 ANTIBODY

A) Activity and specificity of AKR1B10 and AR antibodies

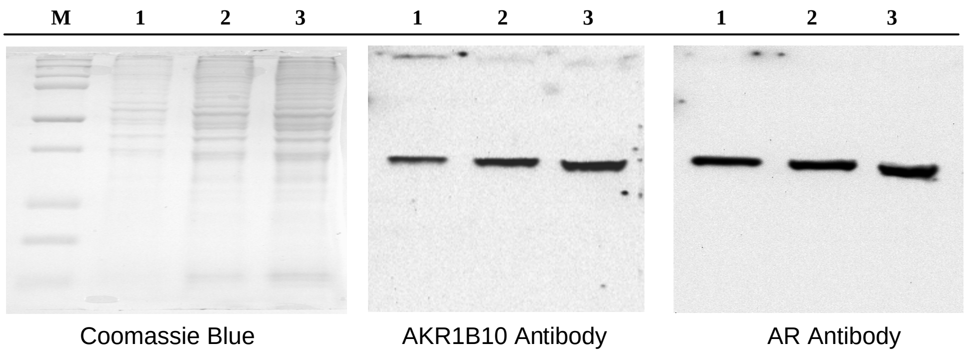

B) Cross-reactivity of AKR1B10 and AR antibodies

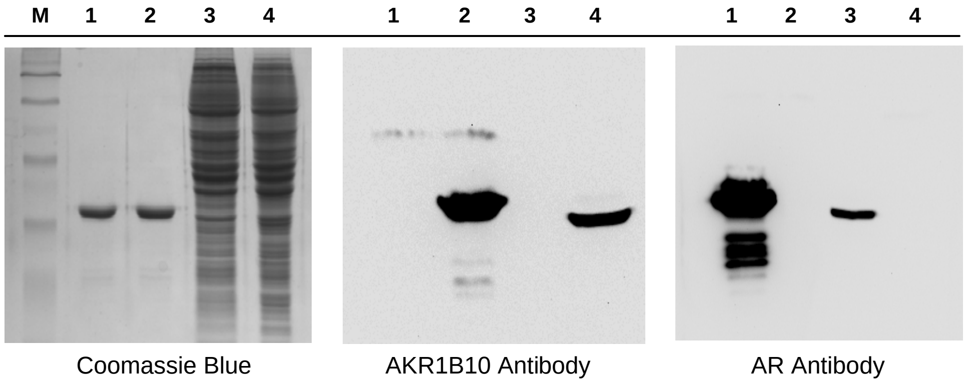

Fig. S1. Activity and specificity of AKR1B10 and aldose reductase (AR) antibodies tested by Western Blot. AKR1B10 and AR antibodies were produced by immunizing rabbits. Antigen sequences are protected by a patent application. *A)* Activity and specificity in whole cell lysates that were prepared by mixing cell suspensions (A549, expressing both AKR1B10 and AR) with an equal volume of 2 x SDS loading buffer and heating at 75°C for 10 min. Lane 1) 1 x 10^4 cells; Lane 2) 5 x 10^4 cells; and Lane 3) 1 x 10^5 cells. *B)* Cross reactivity. AKR1B10 and AR are two close human members in aldo-keto reductase family. Purified AKR1B10 and AR proteins and whole cell lysates were used in this cross-reactivity test. Lane 1) AR protein; Lane 2) AKR1B10 protein; Lane 3) 293T cells, expressing AR; and Lane 4) HCT-8 cells, expressing AKR1B10. Western blot and recombinant protein purification were conducted as described in text; results indicate the high purity of proteins and high specificity of AKR1B10 and AR antibodies.
